# Validation of the ALK-Brain Prognostic Index for patients with ALK-rearranged lung cancer and brain metastases

**DOI:** 10.1016/j.esmoop.2023.102069

**Published:** 2023-11-20

**Authors:** I. Zerdes, C. Kamali, A. Koulouris, M. Elsayed, J. Schnorbach, P. Christopoulos, G. Tsakonas

**Affiliations:** 1 Department of Oncology-Pathology, Karolinska Institutet, Stockholm, Sweden; 2 Thoracic Oncology Center, Karolinska Comprehensive Cancer Center, Karolinska University Hospital, Stockholm, Sweden; 3 Department of Thoracic Oncology, Thoraxklinik, Heidelberg University Hospital, Heidelberg, Germany; 4 Translational Lung Research Center Heidelberg, Member of the German Center for Lung Research (DZL) Heidelberg, Germany

**Keywords:** ALK-BPI, non-small-cell lung cancer, prognosis, brain metastases

## Abstract

**Background:**

Brain metastases (BMs) are a key challenge in the management of anaplastic lymphoma kinase-rearranged non-small-cell lung cancer (ALK+ NSCLC), but prognostic scores are complicated or rely on data before the era of tyrosine kinase inhibitors (TKIs). This study aimed to validate the novel ALK-Brain Prognostic Index (ALK-BPI), which was originally proposed based on 44 TKI-treated ALK+ NSCLC patients from Karolinska University Hospital, using an external clinical cohort.

**Patients and methods:**

TKI-treated ALK+ NSCLC patients with BM from Heidelberg (*n* = 82, cohort 1) were retrospectively analyzed alone and together with the original Karolinska cohort (*n* = 126, cohort 2). Cox regression models were used to determine the association of clinical variables and scores with overall survival (OS) after BM diagnosis (BM-related OS).

**Results:**

Both cohorts showed a similar median age (58 years), roughly balanced sex distributions (52%-56% females), and Eastern Cooperative Oncology Group performance status (PS) 0-2 for most patients (87%-92%) at the time of BM development, which were present already at initial diagnosis in 36%-38% of the patients. Most patients had received next-generation ALK inhibitors (54%-63%), while 55%-56% of patients did not receive any radiotherapy. The ALK-BPI identified poor-risk patients (i.e. featuring ≥ 2/3 risk factors: PS > 2, male sex, development of BM after initial diagnosis) with a significantly shorter BM-related OS than other patients in both cohorts: 32/82 in cohort 1 with 21.3 versus 62.2 months in median [hazard ratio (HR) = 2.5, *P* < 0.001]; 59/126 in cohort 2 with 23.1 versus 67.2 months in median (HR = 2.6, *P* < 0.001). The five-parameter Lung-molGPA score did not achieve statistical significance and/or clear prognostic separation in all four groups, while the Disease-Specific Graded Prognostic Assessment score did not show consistent results.

**Conclusions:**

The ALK-BPI is a reliable tool for easy prognostic dichotomization of TKI-treated ALK+ NSCLC patients with BM in daily clinical practice, without the complexity of previous models.

## Highlights

• BMs are a challenge in the management of ALK+ NSCLC.
• Existing prognostic scores are complex and rely on data before the TKI era.
• ALK-BPI can identify patients with favorable OS based on three clinical variables.
• This validation study shows that ALK-BPI is a reliable prognostic tool for TKI-treated ALK+ NSCLC with BM.

## Introduction

Anaplastic lymphoma kinase-positive (ALK+) non-small-cell lung cancer (NSCLC) represents a paradigm for precision medicine with significant clinical benefit in terms of overall survival (OS) and quality of life from the increasing utilization of tyrosine kinase inhibitors (TKIs) during the past decade.^1^

However, brain metastases (BMs) remain a key challenge in the management of these patients, occurring in ∼30% of newly diagnosed patients with a steady increase over the subsequent disease course up to 70%.^2^ While the introduction of ALK-TKI has provided durable responses and improved intracranial as well as extracranial disease control,^3^ there is still an unmet need to reliably predict the clinical course of brain metastatic disease and guide physicians’ decisions for this heterogeneous patient group. Several different prognostic scores i.e. Recursive Partitioning Analysis (RPA), Graded Prognostic Assessment, and Lung-molGPA have been proposed, including clinical and/or molecular features.^4^, ^5^, ^6^ However, their performance has not been thoroughly evaluated in large cohorts under consideration of targeted therapies, but have rather traditionally emphasized the type of radiotherapy, i.e. whether the patients received stereotactic radiosurgery (SRS) or whole-brain radiotherapy (WBRT).^7^^,^^8^

We have previously proposed a new prognostic score for patients with ALK+ NSCLC and BM [ALK-Brain Prognostic Index (ALK-BPI)], which could identify patients with favorable OS based on clinical variables.^9^ Given its relatively simple formula, potential clinical utility, and comparable performance with other clinical/molecular scores, ALK-BPI could possibly serve as a tool for improved prognostication in daily clinical practice for patients with BMs. Therefore, this study aimed to validate our previously published ALK-BPI in an external clinical cohort of ALK+ NSCLC patients with BMs treated with ALK inhibitors.

## Patients and methods

### Study population and data collection

This retrospective study included 94 consecutive patients with brain metastasized ALK-positive NSCLC who were treated with ALK inhibitors in the Thoraxklinik at Heidelberg University Hospital between February 2009 and July 2021 (cohort 1, external validation cohort), while 12 patients who received chemotherapy upon BM diagnosis were excluded from the study. In addition, these patients were merged with the original Swedish discovery cohort of 44 patients from the Karolinska University Hospital previously described in the original ALK-BPI score publication^9^ and analyzed as a second cohort (cohort 2, merged). ALK positivity was diagnosed using RNA-based next-generation sequencing, as previously described.^9^^,^^10^ Demographic data, histopathology, smoking habits, Eastern Cooperative Oncology Group (ECOG) performance status (PS) at the time of BM diagnosis, number of BMs, presence of extracranial disease, and information about oncologic treatment were collected from the electronic medical records including review of the radiological images by the investigators. Radiologic follow-up was carried out with computerized tomography (CT) of the chest/abdomen and magnetic resonance imaging (MRI) of the brain every 6-12 weeks. The study was approved by the ethical review boards of Karolinska (Dnr 2022-00323-01) and Heidelberg University Hospitals (S-296/2016).

### Prognostic and radiological assessments

ALK-BPI, Lung-molGPA, and Disease-Specific Graded Prognostic Assessment (DS-GPA) scores were calculated for each patient based on the respective clinical and molecular features. ALK-BPI included the following variables: PS, sex, and BM at diagnosis and could separate patients into two prognostic groups, as previously described (Supplementary Table S1, available at https://doi.org/10.1016/j.esmoop.2023.102069): good = 1.5-2.5 points versus poor = 0-1.0 points. Lung-molGPA and DS-GPA prognostic scores classified patients into four groups each according to the following classification/point systems: (i) Lung-molGPA: group 1 = 0-1 points, group 2 = 1.5-2.0 points, group 3 = 2.5-3.0 points, group 4 = 3.5-4.0 points; (ii) DS-GPA groups; group 1 = 0-1.0 points, group 2 = 1.5-2.5 points, group 3 = 3.0, group 4 = 3.5-4.0 points. A brain MRI with contrast was carried out in 74 of the cases while the rest were CT-verified in the validation cohort. In the latter group, three patients had multiple metastases which did not affect the GPA scores. CT scans of the chest and abdomen were carried out within 4 weeks after BM diagnosis to determine the presence of extracranial disease.

### Statistical analysis

Categorical and continuous data were analyzed using descriptive statistics. Univariate and multivariate Cox proportional hazards regression analyses were carried out to determine prognostic variables associated with OS and also evaluate the performance of the various prognostic scores. The estimates are presented in hazard ratios (HRs) with a 95% confidence interval (CI). Various clinical variables are included in the Cox univariate model, such as BMs at diagnosis, sex, PS, age, extracranial metastases, radiotherapy, received treatment, and number of BMs. Variables that were statistically significant, marginally significant, and clinically important were included in the multivariate analysis. Variables included in the ALK-BPI model were also included in the Cox multivariable model. We carried out separate multivariable analyses for the ALK-BPI variables adjusted for received treatment in both the external validation cohort (cohort 1) and the merged cohort (cohort 2). OS was defined from the date of BM diagnosis to the date of death or the last follow-up (BM-related OS). OS was calculated according to Kaplan–Meier (KM) and compared between various groups using the log-rank test. All tests were two-sided, and statistical significance was defined as a *P* value of <0.05. Statistical calculations were carried out using SPSS v27 (IBM Corp, Armonk, NY).

## Results

### Patient characteristics

The median age of patients in the validation cohort 1 was 58 years with a predominance of females (56.1%) and an ECOG PS 0-2 (91.5%). Most patients (58.5%) were never- or light-smokers (<10 pack-years) and approximately one-third had BMs at initial diagnosis (37.8%). All patients were treated with ALK inhibitors in the first line, mostly with next-generation ALK inhibitors (54%), while most of them did not receive any radiotherapy (55%). Similar patient characteristics were observed in the merged cohort 2 which comprised 126 patients as summarized in Table 1. A slightly higher percentage of patients with PS 3-4, patients with more than four brain metastases, and treatment with next-generation ALK inhibitors was noted in the merged dataset as compared to cohort 1 only. A summary of received systemic therapies after the diagnosis of BM, for the subgroup of patients with secondary BM, is presented in Supplementary Table S2, available at https://doi.org/10.1016/j.esmoop.2023.102069.

**Table 1:** Baseline demographics and patient characteristics in cohort 1 (Heidelberg cohort) and cohort 2 (Heidelberg and Karolinska merged cohorts)

Characteristics	Cohort 1 (*n* = 82)	Cohort 2 (*n* = 126)
**Median age, years (range)**	58 (26-83)	58 (26-83)
**Sex, *n*** (%)		
Male	36 (43.9)	60 (47.6)
Female	46 (56.1)	66 (52.4)
**Histology**, *n* (%)		
Adenocarcinoma	79 (96.3)	121 (96.0)
Squamous carcinoma	2 (2.4)	3 (2.4)
Large cell carcinoma	1 (1.2)	2 (1.6)
**ECOG, performance status**, *n* (%)		
0-2	75 (91.5)	109 (86.5)
3-4	7 (8.5)	17 (13.5)
**Smoking history**, *n* (%)		
Never smoker/light smoker	48 (58.5)	75 (59.5)
Ex-smoker	21 (25.6)	37 (29.4)
Smoker (all tobacco)	8 (9.8)	9 (7.1)
Unknown	5 (6.1)	5 (4.0)
**Brain metastases**, *n* (%)		
1-4	46 (56.1)	62 (49.2)
>4	36 (43.9)	64 (50.8)
**Brain metastasis at diagnosis**, *n* (%)		
Yes	31 (37.8)	45 (35.7)
No	51 (62.2)	81 (64.3)
**First-line treatment at BM diagnosis**, *n* (%)		
Crizotinib	38 (46.3)	47 (37.3)
Ceritinib	14 (17.1)	27 (21.4)
Alectinib	14 (17.1)	33 (26.2)
Brigatinib	4 (4.9)	4 (3.2)
Lorlatinib	3 (3.7)	5 (4.0)
No systemic treatment	9 (11.0)	10 (7.9)
**Extracranial metastases**, *n* (%)		
Yes	67 (81.7)	95 (75.4)
No	15 (18.3)	31 (24.6)
**CNS** r**adiotherapy**, *n* (%)		
SRS	12 (14.6)	23 (18.3)
WBRT	25 (30.5)	32 (25.4)
No RT	45 (54.9)	71 (56.3)
**Alive,***n* (%)		
Yes	27 (32.9)	47 (37.3)
No	55 (67.1)	79 (62.7)

BM, brain metastasis; CNS, central nervous system; ECOG, Eastern Cooperative Oncology Group; RT, radiotherapy; SRS, stereotactic radiosurgery; WBRT, whole-brain radiotherapy.

### Clinical factors affecting the prognosis of ALK+ NSCLC with brain metastases

In cohort 1, univariate analysis revealed that primary brain metastatic disease, PS, and age were significantly correlated with OS. Better PS (HR = 0.043, 95% CI 0.014-0.136, *P* < 0.001) was independently associated with longer BM-related OS in multivariable analysis carried out for the ALK-BPI variables, whereas sex (HR = 0.61, 95% CI 0.35-1.06, *P* = 0.082) and primary brain metastatic disease (HR = 0.59, 95% CI 0.33-1.07, *P* = 0.084) showed a strong trend towards longer OS. In the multivariable analysis adjusted for received treatment, all ALK-BPI variables were independently associated with a significant benefit in OS: sex (HR = 0.45, 95% CI 0.24-0.84, *P* = 0.012), primary brain metastatic disease (HR = 0.48, 95% CI 0.25-0.94, *P* = 0.033), and PS (HR = 0.18, 95% CI 0.0.031-0.99, *P* = 0.049) (Table 2).

**Table 2:** Univariate and multivariate Cox proportional hazards regression analyses of variables associated with overall survival in both patient cohorts

Univariate analysis	Cohort 1 (*n* = 82)		Cohort 2 (*n* = 126)	
Variables	Hazard ratio (95% CI)	*P* value	Hazard ratio (95% CI)	*P* value
Brain metastases (primary **versus** secondary)	0.47 (0.27-0.84)	**0.010**	0.38 (0.23-0.63)	**<0.001**
Sex (female **versus** male)	0.69 (0.41-1.18)	0.173	0.58 (0.37-0.91)	**0.018**
PS (<3 versus 3-4)	0.045 (0.015-0.132)	**<0.001**	0.27 (0.15-0.50)	**<0.001**
Age	1.03 (1.00-1.05)	**0.029**	1.03 (1.01-1.05)	**0.004**
Extracranial metastases (yes versus no)	2.11 (0.90-4.92)	0.086	0.47 (0.26-0.85)	**0.012**
Radiotherapy (yes versus no)	1.02 (0.60-1.73)	0.951	1.09 (0.70-1.70)	0.708
Brain metastases, no (1-4 versus >4)	0.83 (0.49-1.42)	0.501	0.77 (0.49-1.19)	0.238
**Treatment at BM**				
Crizotinib	1		1	
No treatment	20.48 (6.93-60.51)	**<0.001**	15.56 (6.16-39.33)	**<0.001**
Lorlatinib	6.56 (1.39-30.80)	**0.017**	1.08 (0.33-3.54)	0.895
Ceritinib	0.87 (0.41-1.86)	0.725	0.82 (0.46-1.47)	0.506
Alectinib	0.70 (0.29-1.74)	0.445	0.57 (0.30-1.12)	0.102
Brigatinib	0.66 (0.16-2.78)	0.571	0.59 (0.14-2.46)	0.469
**Multivariable analysis of the ALK-BPI parameters**				
Brain metastases (primary versus secondary)	0.48 (0.25-0.94)	**0.033**	0.38 (0.22-0.66)	**<0.001**
Sex (female versus male)	0.45 (0.24-0.84)	**0.012**	0.47 (0.29-0.76)	**0.002**
PS (<3 versus 3-4)	0.18 (0.0.031-0.99)	**0.049**	0.30 (0.14-0.65)	**0.003**
Crizotinib	1		1	
No treatment	4.77 (0.90-25.31)	0.067	5.54 (2.01-15.22)	**<0.001**
Lorlatinib	4.32 (0.89-20.84)	0.069	0.55 (0.16-1.85)	0.331
Ceritinib	0.39 (0.16-0.95)	**0.039**	0.43 (0.23-0.82)	**0.010**
Alectinib	0.78 (0.31-1.95)	0.599	0.38 (0.18-0.79)	**0.009**
Brigatinib	0.34 (0.076-1.51)	0.156	0.31 (0.072-1.32)	0.113

Age, as a continuous variable.

Statistically significant values are marked with bold typeface.

ALK-BPI, Anaplastic Lymphoma Kinase-Brain Prognostic Index; BM, brain metastases; CI, confidence interval; PS, performance score.

In the merged cohort 2, the aforementioned clinical variables and also sex and absence of extracranial metastases were found to be prognostic for OS. All ALK-BPI variables, i.e. female sex (HR = 0.59, 95% CI 0.37-0.92, *P* = 0.02), primary brain metastatic disease (HR = 0.42, 95% CI 0.25-0.70, *P* < 0.001), and PS (HR = 0.32, 95% CI 0.17-0.59, *P* < 0.001) remained significant independent prognostic factors of OS in the multivariable model carried out for the ALK-BPI variables. The aforementioned variables sustained the statistically significant correlation with OS in the multivariable analysis adjusted for received treatment: sex (HR = 0.47, 95% CI 0.29-0.76, *P* = 0.002), primary brain metastatic disease (HR = 0.38, 95% CI 0.22-0.66, *P* < 0.001), and PS (HR = 0.30, 95% CI 0.14-0.65, *P* = 0.003) (Table 2).

### Prognostic performance of ALK-BPI and comparison with other clinical scores

**Figure 1:**
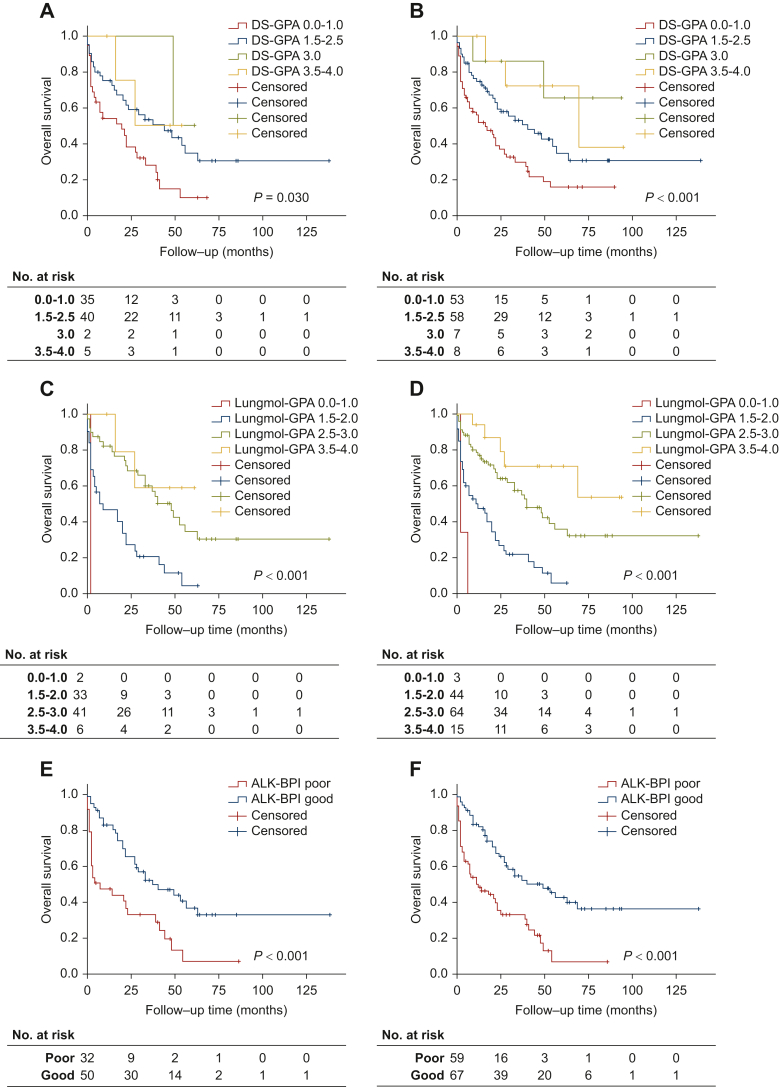
Kaplan–Meier survival estimates for the DS-GPA (A and B), Lung-molGPA (C and D), and ALK-BPI (E and F) in cohort 1 (Heidelberg cohort, upper panel) and in cohort 2 (Heidelberg and Karolinska merged cohorts, lower panel). ALK-BPI, Anaplastic Lymphoma Kinase-Brain Prognostic Index; DS-GPA, Disease-Specific Graded Prognostic Assessment.

Both DS-GPA and Lung-molGPA classified patients into four groups with group 1 being associated with the worst prognosis. Although the magnitude of survival benefit was generally proportional to the classification group, not all DS-GPA and/or Lung-molGPA groups differed significantly in the univariate analysis. While the lowest group of DS-GPA showed significantly worse survival in both cohorts, the other three groups did not show consistent separation. Furthermore, although not all Lung-molGPA groups achieved statistical significance, the lower-graded groups showed consistently worse prognoses in both cohorts (Tables 3 and 4). The absolute difference in mOS between the two prognostic groups of the ALK-BPI was more profound and consistent in both the Heidelberg and merged cohorts (62.2 versus 21.3 months in the Heidelberg cohort, 67.2 versus 23.1 months in the merged). The differences in mOS in the four prognostic groups in the DS-GPA and Lung-molGPA were not equally profound and some groups showed similar mOS (Tables 3 and 4) The KM curves for the four-tier DS-GPA and Lung-molGPA models between the groups and OS are shown in Figure 1A-D. Regarding the ALK-BPI score, the patients were classified into two groups based on the scoring of the model’s variables (PS, sex, and BM at diagnosis), as group 1 (‘high score’), with a median OS (mOS) of 62.2 months (95% CI 45.4-79.0 months), and group 2 (‘low score’), with an mOS of 21.3 months (95% CI 12.0-30.6 months) in cohort 1. The ALK-BPI low-score group was significantly associated with worse BM-related OS both in the validation (HR_uni_ = 2.5, 95% CI 1.4-4.2, *P* < 0.001) and in the merged cohorts (HR_uni_ = 2.6, 95% CI 1.7-4.1, *P* < 0.001) (Tables 3 and 4). Furthermore, a significant difference between the good and poor ALK-BPI groups was demonstrated in the separation of KM curves with the pairwise log-rank test in both cohorts (log-rank *P* < 0.001, Figure 1E and F).

**Table 3:** Univariate Cox regression overall survival analysis of DS-GPA, Lung-molGPA, and ALK-BPI in cohort 1 (Heidelberg cohort)

	Patients, n (%)	mOS in months (95% CI)	Hazard ratio (95% CI)	*P* value
**DS-GPA**				
Group 4 (3.5-4.0)	5 (6.1)	37.8 (21.4-54.1)	0.3 (0.08-1.4)	0.142
Group 3 (3.0)	2 (2.4)	55.0 (46.7-63.3)	0.3 (0.03-1.9)	0.177
Group 2 (1.5-2.5)	40 (48.8)	58.9 (40.0-77.6)	0.5 (0.3-0.9)	**0.013**
Group 1 (0-1.0)	35 (42.7)	22.2 (14.7-29.8)	1	
**Lung-molGPA**				
Group 4 (3.5-4.0)	6 (7.3)	45.2 (28.0-62.4)	1	
Group 3 (2.5-3.0)	41 (50.0)	64.1 (45.8-82.5)	1.7 (0.4-7.3)	0.467
Group 2 (1.5-2.0)	33 (40.2)	18.8 (11.8-25.8)	4.9 (1.1-20.5)	**0.032**
Group 1 (0-1.0)	2 (2.4)	2.0 (2.0-2.0)	15.5 (2.1-117.2)	**0.008**
**ALK-BPI**				
Good (1.5-2.5)	50 (61.0)	62.2 (45.4-79.0)	1	
Poor (0-1.0)	32 (39.0)	21.3 (12.0-30.6)	2.5 (1.4-4.2)	**<0.001**

The numbers in the different prognostic groups indicate the point scoring system in each group.

Statistically significant values are marked with bold typeface.

ALK-BPI, Anaplastic Lymphoma Kinase-Brain Prognostic Index; DS-GPA, Disease-Specific Graded Prognostic Assessment; CI, confidence interval; mOS, median overall survival.

**Table 4:** Univariate Cox regression overall survival analysis of DS-GPA, Lung-molGPA, and ALK-BPI in cohort 2 (Heidelberg and Karolinska merged cohorts)

	Patients, *n* (%)	mOS in months (95% CI)	Hazard ratio (95% CI)	*P* value
**DS-GPA**				
Group 4 (3.5-4.0)	8 (6.3)	64.4 (40.7-88.0)	1	
Group 3 (3.0)	7 (5.6)	71.6 (46.8-96.4)	0.7 (0.1-4.3)	0.724
Group 2 (1.5-2.5)	58 (46.0)	56.7 (40.3-73.2)	2.0 (0.6-6.7)	0.238
Group 1 (0-1.0)	53 (42.0)	24.9 (16.7-33.2)	3.9 (1.2-12.7)	**0.023**
**Lung-molGPA**				
Group 4 (3.5-4.0)	15 (11.9)	67.7 (49.4-85.9)	1	
Group 3 (2.5-3.0)	64 (50.8)	61.8 (46.1-77.6)	2.1 (0.8-5.3)	0.123
Group 2 (1.5-2.0)	44 (34.9)	18.3 (12.4-24.2)	5.8 (2.2-14.9)	**<0.001**
Group 1 (0-1.0)	3 (2.4)	3.3 (0.7-5.9)	17.8 (4.0-79.0)	**<0.001**
**ALK-BPI**				
Good (1.5-2.5)	67 (53.2)	67.2 (52.1-82.4)	1	
Poor (0-1.0)	59 (46.8)	23.1 (15.8-30.3)	2.6 (1.6-4.1)	**<0.001**

	**Patients,n(%)**	**mOS in months (95% CI)**	**Hazard ratio (95% CI)**	***P* value**
**DS-GPA**				
Group 4 (3.5-4.0)	8 (6.3)	64.4 (40.7-88.0)	1	
Group 3 (3.0)	7 (5.6)	71.6 (46.8-96.4)	0.7 (0.1-4.3)	0.724
Group 2 (1.5-2.5)	58 (46.0)	56.7 (40.3-73.2)	2.0 (0.6-6.7)	0.238
Group 1 (0-1.0)	53 (42.0)	24.9 (16.7-33.2)	3.9 (1.2-12.7)	**0.023**
**Lung-molGPA**				
Group 4 (3.5-4.0)	15 (11.9)	67.7 (49.4-85.9)	1	
Group 3 (2.5-3.0)	64 (50.8)	61.8 (46.1-77.6)	2.1 (0.8-5.3)	0.123
Group 2 (1.5-2.0)	44 (34.9)	18.3 (12.4-24.2)	5.8 (2.2-14.9)	**<0.001**
Group 1 (0-1.0)	3 (2.4)	3.3 (0.7-5.9)	17.8 (4.0-79.0)	**<0.001**
**ALK-BPI**				
Good (1.5-2.5)	67 (53.2)	67.2 (52.1-82.4)	1	
Poor (0-1.0)	59 (46.8)	23.1 (15.8-30.3)	2.6 (1.6-4.1)	**<0.001**

The numbers in the different prognostic groups indicate the point scoring system in each group.

Statistically significant values are marked with bold typeface.

ALK-BPI, Anaplastic Lymphoma Kinase-Brain Prognostic Index; CI, confidence interval; DS-GPA, Disease-Specific Graded Prognostic Assessment; mOS, median overall survival.

## Discussion

BMs remain an important challenge in the management of patients with ALK+ NSCLC.^1^^,^^11^ Hence, there is a need for reliable tools and biomarkers to estimate the prognosis of patients with BM, especially in the targeted oncology era. Towards this end, this retrospective cohort study aimed to validate the previous promising results of the novel prognostic score ALK-BPI using high-quality real-world data from two large European academic thoracic oncology centers, namely the Thoraxklinik at Heidelberg University Hospital in Germany and Karolinska University Hospital in Sweden. Initially, this index was developed and tested in a single-center Swedish cohort including 44 ALK+ NSCLC patients with BM who received ALK-TKIs as first-line treatment.^9^ This index could divide patients into two prognostic groups based on the following three factors: PS, sex, and the presence of BM at diagnosis. The present study demonstrated the capacity of the ALK-BPI model to significantly predict BM-related OS in an external cohort almost twice as large as the discovery cohort of the original publication, and the effect was also maintained after the two cohorts were merged.

ALK-BPI represents the first—to our knowledge—prognostic score developed for ALK+ NSCLC patients with BM who were treated solely with targeted therapies in the first-line setting. Given that all patients in both cohorts received treatment with ALK inhibitors, this prognostic index is therefore validated in this particular population. Although a plethora of prognostic indices have been proposed, not all patients included have received targeted therapies and have yet to be tested in large cohorts of oncogene-addicted NSCLC patients. Indeed, the majority of prognostic scores have been developed in patients receiving radiotherapy such as the RPA, the basic score for brain metastases, the score index for stereotactic radiosurgery of brain metastases (SIR), or the comprehensive prognostic index.^4^, ^5^, ^6^^,^^12^^,^^13^ The introduction of GPA index, DS-GPA, and recently the Lung-molGPA has gradually paved the way for the incorporation of molecular features (epidermal growth factor receptor and/or ALK) to clinical variables, but still lack validation in large multicenter studies with only ALK+ NSCLC patients.^13^

In this study, we calculated DS-GPA and Lung-molGPA scores in patients treated with ALK inhibitors and we observed that their prognostic information was preserved, while both scores presented with an equivalent prognostic value as the ALK-BPI. However, the absolute difference in mOS in months was not so clear between the different prognostic groups of the Lung-molGPA and DS-GPA scoring systems, something which could question the need for four prognostic groups for ALK+ NSCLC patients with BM, instead of two, as in the ALK-BPI. Moreover, unlike the aforementioned scores, the two-tier approach proposed with ALK-BPI renders the score calculation and patient classification simpler in the clinical routine. Of note, a clinically and statistically significant consistent absolute difference of 40.9 months and 44.1 months in median OS between the two ALK-BPI groups was observed in cohort 1 and cohort 2, respectively.

The main limitation of the current study is its retrospective nature, which cannot exclude the presence of potential confounders. On the other hand, the reproducibility of the results in an independent cohort argues for the validity and generalizability of our findings. Nonetheless, the overall sample size is relatively small with considerable risk for type 2 statistical errors, which could explain the marginal statistical significance of the two ALK-BPI variables (sex and primary brain metastatic disease) in the non-adjusted for received treatment multivariable analysis carried out in the Heidelberg cohort. The latter finding could also be attributed to the higher proportion of patients treated with crizotinib in the Heidelberg cohort compared to the Karolinska cohort; the worse intracranial clinical benefit of first-generation ALK-TKI crizotinib versus second- and third-generation TKIs, especially in the primary brain metastatic setting, could also possibly explain the minor discrepancies observed in the Heidelberg multivariate analysis which only included the ALK-BPI variables.^14^, ^15^, ^16^ When the multivariable analysis was adjusted for received treatment, all ALK-BPI variables were correlated with a statistically significant longer OS. This latter finding suggests the received TKI as the reason behind the minor discrepancies observed in the non-adjusted for treatment multivariate analysis carried out in cohort 1. All ALK-BPI variables showed a statistically significant longer mOS in both the univariate and multivariate (both non-adjusted and adjusted for received treatment) analyses carried out in the merged cohort. The treatments that patients have received after first-line systemic therapy for brain metastatic disease can also affect OS. Specifically, we observed a relatively high dropout rate between subsequent lines of therapy, similar to previous reports,^17^ in patients with secondary BM, albeit the limited sample size did not allow further statistical analyses regarding treatment sequence (Supplementary Table S2, available at https://doi.org/10.1016/j.esmoop.2023.102069).

Our findings underscore the need for prospective and multicentric validation of the proposed prognostic score, in particular under the inclusion of more patients treated with the newer next-generation ALK-TKIs that have improved brain efficacy and are preferred for the initial patient treatment currently.^3^^,^^18^^,^^19^ One future step could also be the enrichment of prognostic algorithms with molecular tumor features, such as the presence of the shorter and more oncogenic *EML4-ALK* fusion variant 3, which has been associated with earlier intracranial progression in TKI-treated ALK+ NSCLC.^10^

In conclusion, this validation study including patients from two large academic centers demonstrated that ALK-BPI is a novel prognostic tool for TKI-treated ALK+ NSCLC patients with BM, and thus represents a practical method for refined patient stratification in daily clinical practice.
