# Supplementary material for: Validation of the ALK-Brain Prognostic Index for patients with ALK-rearranged lung cancer and brain metastases
Source: ESMO Open. 2023 Nov 20;8(6):102069. doi: 10.1016/j.esmoop.2023.102069 (PMC10774967; doi:10.1016/j.esmoop.2023.102069)
Supplement: Supplementary Table 1 [file mmc1.docx]

| **Prognostic factor** | **Scoring criteria** | | |
| --- | --- | --- | --- |
|  | 0 | 0.5 | 1 |
| Brain metastasis at diagnosis | No | - | Yes |
| Performance status | 3-4 | 2 | 0-1 |
| Sex | Male | Female | - |
