# Supplementary material for: Validation of the ALK-Brain Prognostic Index for patients with ALK-rearranged lung cancer and brain metastases
Source: ESMO Open. 2023 Nov 20;8(6):102069. doi: 10.1016/j.esmoop.2023.102069 (PMC10774967; doi:10.1016/j.esmoop.2023.102069)
Supplement: Supplementary Table 2 [file mmc2.docx]

|  | | | | Cohort 1 (N=51)  n (%) of overall population) | | Cohort 2 (N=81)  n (%) of overall population) | |
| --- | --- | --- | --- | --- | --- | --- | --- |
| 1st Line  Crizotinib  Ceritinib  Alectinib  Brigatinib  Lorlatinib  No systemic treatment | | | | 42 (82.4)  17 (33.3)  13 (25.5)  6 (11.8)  3 (5.9)  3 (5.9)  9 (17.6) | 71 (87.7)  23 (28.4)  21 (25.9)  19 (23.5)  3 (3.7)  5 (6.2)  10 (12.3) | | |
|  | | | |  |  | |  |
| 2nd Line  Ceritinib  Alectinib  Brigatinib  Lorlatinib  Chemotherapy  Immunotherapy  No systemic treatment | | | | 23 (45.1)  7 (13.7)  10 (19.6)  0 (0.0)  4 (7.8)  1 (2.0)  1 (2.0)  19 (37.3) | 32 (39.5)  8 (9.9)  12 (14.8)  2 (2.5)  7 (8.6)  2 (2.5)  1 (1.2)  39 (48.1) | | |
|  | | | |  |  | |  |
| 3rd Line  Ceritinib  Alectinib  Brigatinib  Lorlatinib  Chemotherapy  No systemic treatment | | | | 13 (25.5)  1 (2.0)  3 (5.9)  3 (5.9)  2 (3.9)  4 (7.8)  10 (19.6) | 17 (21.0)  1 (1.2)  5 (6.2)  3 (3.7)  3 (3.7)  5 (6.2)  15 (18.5) | | |
|  |  |  |  |  |  |  |  |
| 4th Line  Alectinib  Brigatinib  Lorlatinib  No systemic treatment | | | | 4 (7.8)  1 (2.0)  0 (0.0)  3 (5.9)  9 (17.6) | 7 (8.6)  1 (1.2)  1 (1.2)  5 (6.2)  10 (12.3) | | |
|  | | | |  |  | |  |
| 5th Line  Brigatinib  Chemotherapy  Immunotherapy  No systemic treatment | | | | 1 (2.0)  0 (0.0)  0 (0.0)  1 (2.0)  3 (5.9) | 4 (4.9)  2 (2.5)  1 (1.2)  1 (1.2)  3 (3.7) | | |
|  | | | |  |  | |  |
| 6th Line  Crizotinib  Chemotherapy  No systemic treatment | | | | 1 (2.0)  0 (0.0)  1 (2.0)  0 (0.0) | 2 (2.5)  1 (1.2)  1 (1.2)  2 (2.5) | | |
